# Recommendations from a Dialogue on Evolving National Cancer Institute-Designated Comprehensive Cancer Center Community Outreach and Engagement Requirements: A Path Forward

**DOI:** 10.1089/heq.2020.0156

**Published:** 2021-02-26

**Authors:** Patricia M. Doykos, Moon S. Chen, Karriem Watson, Vida Henderson, Monica L. Baskin, Sarah Downer, Lauren A. Smith, Neeraja Bhavaraju, Samantha Dina, Christopher S. Lathan

**Affiliations:** ^1^ Bristol Myers Squibb Foundation, New York, New York, USA.; ^2^ UC Davis Comprehensive Cancer Center, Sacramento, California, USA.; ^3^ University of Illinois Cancer Center, Chicago, Illinois, USA.; ^4^ O'Neal Comprehensive Cancer Center, University of Alabama at Birmingham, Birmingham, Alabama, USA.; ^5^ The Center for Health Law and Policy Innovation, Harvard Law School, Cambridge, Massachusetts, USA.; ^6^ FSG, Boston, Massachusetts, USA.; ^7^ Dana-Farber Harvard Cancer Center, Boston, Massachusetts, USA.

**Keywords:** cancer center, cancer institute, community outreach and engagement

## Abstract

While cancer mortality is declining in the United States, significant racial, ethnic, economic and geographic inequities persist. To help address inequities in cancer treatment, care, support and research, the National Cancer Institute (NCI) instituted the community outreach and engagement (COE) mandate for NCI-designated comprehensive cancer centers (CCCs). The Bristol Myers Squibb Foundation designed a convening and listening session on COE with NCI leaders and staff gathering representatives from CCCs and the broader cancer community. This paper captures recommendations from the listening session for the NCI and CCCs to further evolve the implementation and impact of the COE mandate on cancer control and outcomes.

While cancer mortality is declining in the United States, significant racial, ethnic, economic, and geographic inequities persist. A number of factors inside and outside of clinical care contribute to these disparities, including environmental, socioeconomic, and biologic factors, as well as historic distribution of the structural and social determinants of health (SDOH).^1^ The level of institutional commitment and capacity of cancer research and care organizations to effectively engage with and serve disproportionately affected and medically underserved populations have also contributed to these inequities. Yet today, with a confluence of factors, including scientific breakthroughs in cancer prevention, detection, and treatment, demographic trends toward growing racial and ethnic minority populations, growing cancer burden among these and rural populations, and reignited social justice and equity movements, mitigating these inequities has never been more critical.^2,3^

In recent years, the cancer research and care community has been more attuned to health equity and has pursued increasingly coordinated and comprehensive action. For example, the American Association for Cancer Research (AACR), the American Cancer Society (ACS), the American Society of Clinical Oncology (ASCO), and the National Cancer Institute (NCI) issued a joint position statement on the research agenda for health disparities in 2017.^4^ The Centers for Disease Control's (CDC) National Comprehensive Cancer Control Program also cites health equity as a cross-cutting priority and highlights three areas for action: training a culturally competent workforce, promoting equitable access to resources, and using data measurement in research and surveillance to guide community-driven plans.^5^

The NCI has recently started to shift more fully in this direction with its 2012 Cancer Center Support Grants (CCSG) requirement for cancer centers to define and address the needs of a local “catchment area” (CA) and the 2016 mandate for Community Outreach and Engagement (COE).^6^ The COE mandate identifies seven areas of action for NCI-designated Comprehensive Cancer Centers (CCCs): (1) defining a CA and understanding the CA population; (2) performing research to address the needs of the CA population; (3) engaging the CA population; (4) taking action to address cancer disparities in the CA; (5) designing clinical trials to represent the diversity of the CA population; (6) translating research into policy recommendations; and (7) extending research and policy within and beyond the CA.^7^

While these mandates reference disparities and encourage CCC activity to address them, there remains significant opportunity to improve the effectiveness and impact of COE on cancer disparities. At a special convening in April 2019, the Bristol Myers Squibb Foundation (BMSF) hosted a dialogue between the NCI, 22 NCI-designated, emerging, and affiliated CCCs, and affiliated programs, and the broader cancer community to understand the current state of COE and identify opportunities to improve COE and cancer health equity efforts more broadly. NCI co-developed the agenda with BMSF. Presentations highlighted that early experience with COE points to several adjustments that could inform ongoing revisions to the CCSG guidelines, including a more explicit focus on developing and maintaining high quality, authentic engagement with the community, deeper understanding of the historic and current structural barriers that contribute to inequities and how to address them, and effective resourcing and evaluation of COE as a tool to improve equity. This article shares highlights of COE work across NCI CCCs and major recommendations that emerged from the meeting discussion for the NCI and for cancer centers to deepen their impact on cancer inequities.

## Implementation Experience with COE Across NCI-Designated, Affiliated, and Emerging CCCs

**FIG. 1.**
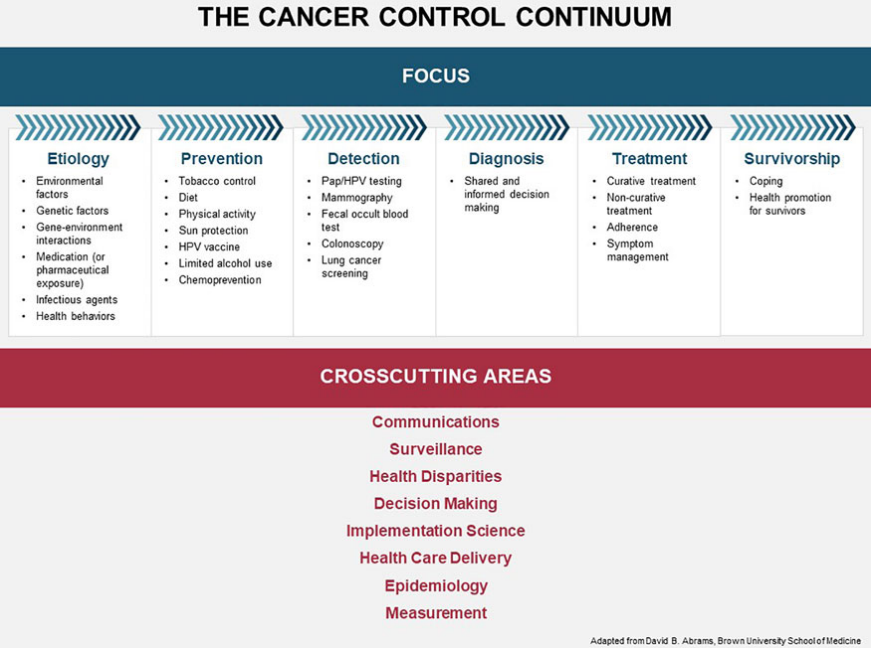
The Cancer Control Continuum.

Inequitable health outcomes are the result of historical and structural issues that have created and perpetuated disparities across every stage and aspect of cancer care, from risk factors to diagnosis, treatment, and survivorship. The NCI Cancer Control Continuum (Fig. 1) captures these factors and provides a comprehensive framework to consider the various goals and activities for COE.^8^ Convening participants shared examples of their work to address disparities across the Cancer Control Continuum, summarized in Table 1.

**Table 1.** Summary of Common Community Outreach and Engagement Interventions Along the Cancer Control Continuum

Phase	COE goals	Examples of COE interventions
Etiology and Research	Conduct research to improve understanding of cancer inequities and how to address them	• *Patient segmentation*: Use of data such as patient residence zip code, income, race, and ethnicity to detect risk factors to be addressed in care
		• *Community research*: Qualitative research (e.g., patient focus groups) to understand structural and social barriers to accessing prevention and care
		• *Community advisory structures*: Formal structures that foster trusting relationships between cancer centers and their communities and ensure that cancer center research is informed by the needs and interests of the community
		• *Clinical trial diversity*: Relationship-building and recruitment to clinical trials to ensure representation that reflects the diverse end-user populations
Prevention	Address inequities in risk factors for cancer	• *Community screening and education*: Partnerships between cancer centers and community organizations to engage local communities on prevention education, behavior change support, and screening (e.g., with patient navigators)
		• *Policy advocacy*: Lends expertise to and collaborates with existing coalitions and other efforts to advocate for effective policy or regulation to mitigate disparities in exposure to cancer risk factors within the community
Detection and Diagnosis	Mitigate disparities in the quality and timeliness of cancer screening and diagnosis	• *Health system partnerships*: Expansion of access to cancer center diagnosis expertise through partnerships with local clinics and/or telementoring, for example, Project ECHO or other training for primary care providers
		• *Language and culture-appropriate care*: Translation services to provide language-appropriate care throughout catchment area
		• *Shared decision making*: Incorporation of patient perspectives, values, and preferences at the time of diagnosis to inform care decisions
		• *Community and government partnerships*: Structural changes to align priorities, resources, and action to improve prevention and early detection for cancer
Treatment	Reduce disparities in treatment outcomes by improving the consistency and quality of cancer care	• *Streamlined care pathways*: Updates to health care delivery procedures and supports to enable easier navigation of complex courses of care
		• *Partnerships with nonhealth service providers*: Holistic care that enables people to complete courses of care effectively, which could include partnerships with social support services to address housing, food, and other needs
Survivorship	Promote high-quality support and care for individuals after treatment	• *Health promotion education*: Health promotion education for survivors, especially those from communities experiencing disproportionate incidence and mortality
		• *Effective post-clinical transition*: Capacity building for local providers to support survivor transitions from specialty to primary and community care settings
Cross-cutting areas	Improve the delivery of cancer care across all stages of the continuum	• *Evidence-based practices*: Disseminate EBPs and tools to providers and community organizations within catchment area to assist in implementation
		• *Communication through telementoring*: Strengthening of connections between providers within cancer care system to improve care across the catchment area

COE, Community Outreach and Engagement; EBP, evidence-based practice.

## Recommendations for Developing, Delivering, and Supporting Impactful COE

**FIG. 2.**
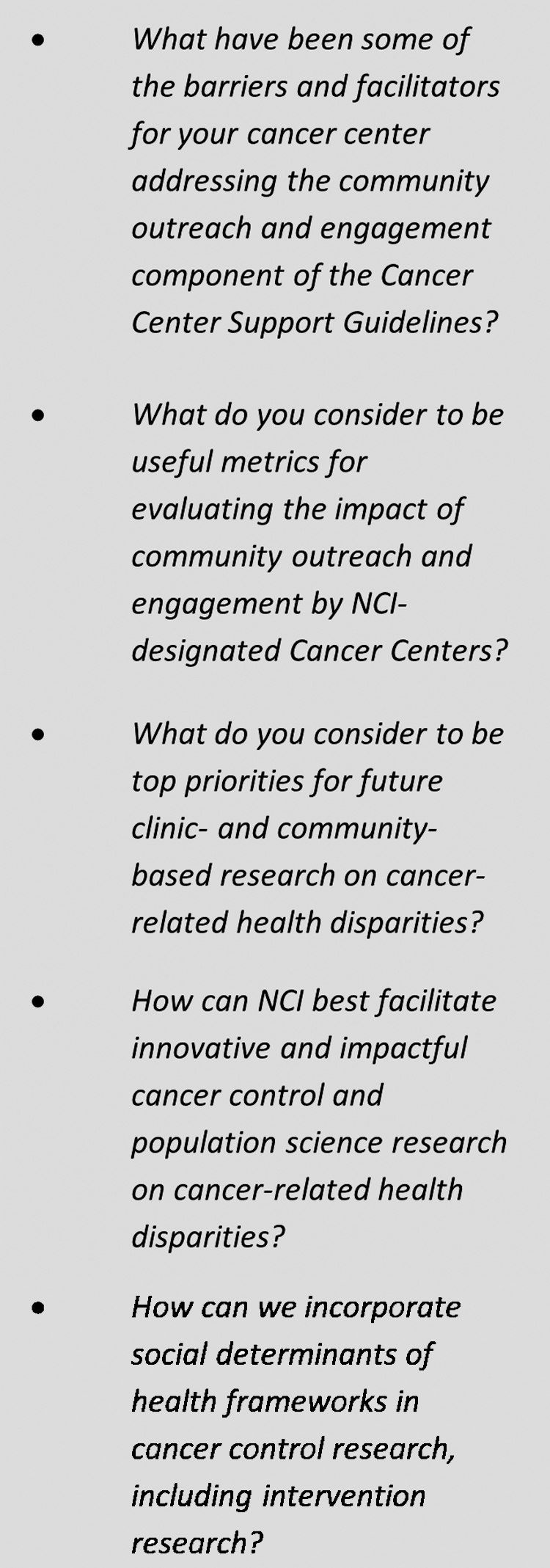
NCI COE listening session questions. COE, Community Outreach and Engagement; NCI, National Cancer Institute.

Many cancer centers are actively implementing COE efforts across the Cancer Control Continuum in an effort to reduce cancer disparities. However, while these individual COE interventions are demonstrating impact, they will need to be sustained, scaled, and deeply tied to a systemic health equity approach to meaningfully impact cancer burden.^9^ In response to questions that NCI prepared for the listening session (Fig. 2), the meeting discussion surfaced five major opportunities to improve the impact of COE.

### Adopt and adequately resource an explicit health equity approach

For COE efforts to result in meaningful and equitable improvement in access to care, quality of care, and cancer outcomes, they must target inequities along the Cancer Control Continuum (Fig. 2). While the NCI COE mandate acknowledges the unique role cancer centers can play within their communities and provides some resources for that work, further resourcing and explicit focus on heath equity will help cancer centers prioritize those COE efforts with the highest potential for impact.^10^

#### Recommendations for NCI

1. Integrate language specifically for populations experiencing health disparities and for equity in health outcomes into the COE mandate.
2. Support research to identify multilevel drivers of cancer disparities in CAs and implementation science to identify and build the investment case for scaling effective COE practices.
3. Support cancer centers to use their unique positions in the community to encourage public–private partnerships to attract additional resources to support innovative COE pilot projects or to scale effective COE practices.

#### Recommendations for cancer centers

1. Increase funding for COE efforts as a more substantial portion of core NCI grants.
2. Create accountability structures and common measures of success around inclusive research and health equity to ensure it is embedded across departments and functions.^11^
3. Establish and utilize a community advisory board to create alignment between research priorities and community needs.
4. Conduct COE pilots and track results to inform improved implementation and make the case for sustaining, scaling, or replicating pilot interventions.

### Understand and address structural barriers to equitable cancer outcomes

A critical step in developing COE interventions that effectively address health inequities is to understand the complex factors that influence a patient's ability to effectively engage in cancer care, including impacts of structural violence and racism, environmental factors, and access to resources and support services. For example, a primary component of the NCI COE mandate is to increase diversity in clinical trials. While seeking diverse and representative clinical trial participation is essential from a scientific perspective, research demonstrates that a history of slavery, medical exploitation, and structural racism has prevented African Americans from participating in medical research.^12^ Without addressing these factors, cancer centers are limited in their ability to effectively pursue diversity in clinical trials and provide effective and inclusive care for their local communities.

#### Recommendations for NCI

1. Shift from a narrow focus on clinical trial recruitment to a longer-term focus on addressing the challenges experienced by the community along the Cancer Control Continuum that interfere with participation in trials and treatment. For example, support not only recruitment of diverse populations into clinical trials but also provision of transportation, childcare, or other social support to enable successful completion of clinical trials among diverse populations.
2. Evaluate cancer centers on their uptake of best practices to address structural determinants of health (e.g., requiring centers to develop systematic, comprehensive screening and service connection efforts to identify SDOH challenges, and bridge the gap to community resources).
3. Fully integrate existing research and recommendations on health inequities and evidence-based practices into NCI CCSG guidelines to help cancer centers improve COE efforts, including effective strategies developed by other public health entities, including other branches of the NIH and the CDC.^13^

#### Recommendations for cancer centers

1. Embed systematic screening for relevant SDOH factors in patient care and demographically segment patient data to uncover health disparities in diagnosis, care outcomes, and access pathways, and understand how SDOH factors impact risk factors and health outcomes for local populations.
2. Establish long-term partnerships with trusted community organizations that can help build understanding of local communities, establish credibility and trust, and connect patients with nonclinical resources.

### Improve access to and quality of care by streamlining patient pathways across clinical settings:

Silos between CCCs and other clinical settings in communities (i.e., primary care providers, federally qualified health centers, safety net hospitals, and community oncology) limit the ability of CCCs to engage effectively in their catchment areas. This becomes an even larger challenge when CCCs seek to change systemic conditions in their local communities, which requires collaboration with actors both within and outside of the health sector. While some cancer centers have mitigated these challenges by establishing one-off partnerships with other health care organizations and systems, greater change is needed to enable more significant, sustainable partnerships for COE. Engaging public and private payers and health plans is also critical to sustainability.

#### Recommendations for NCI

1. Provide resources for partnership or coalition development and maintenance to promote and support collaboration between cancer centers, or between cancer centers and other health and nonhealth systems addressing SDOH. For example, CRCHD U54s are a helpful mechanism to support mentorship and relationships between institutions for research. A similar mechanism could support collaboration to expand access to cancer screening and treatment.
2. Provide resources that cancer centers can share with community partners to build trust and demonstrate respect for the time, effort, and connections community partners provide.
3. Require cancer centers to report current or potential future partnerships they will pursue to address health equity along with a plan for addressing power dynamics.

#### Recommendations for cancer centers

1. Establish partnerships with other health systems to collectively address major risk factors and structural determinants of health relevant for shared catchment areas.
2. Build capacity of local partners to deliver high-quality, up-to-date care, diagnosis, and referral services at primary care sites that are more accessible to populations experiencing health inequities.

### Advance relevant policies that support cancer control

A number of city, state, and federal policies influence the context in which cancer centers operate by decreasing risk factors and increasing protective factors for cancer. For example, over 50 communities,^14^ including the city of San Francisco and the Commonwealth of Massachusetts, have banned the sale of menthol cigarettes, which have been specifically targeted to communities of color. Organizations like the African American Tobacco Control Leadership Council (AATCLC) and National Association for the Advancement of Colored People are backing these efforts, and adding further support is one example of how the NCI can seek more equitable cancer outcomes through policy.

More broadly, equitable cancer outcomes require universal health care. Our current system of employer-sponsored private health insurance for working adults between 26 and 65 years of age is fundamentally flawed since illness, especially symptomatic cancer, can often lead to job loss and loss of health care coverage. While many cancer centers are engaging local populations on early screening and diagnosis, the lack of treatment coverage is a major barrier to establishing sustainable mechanisms for linkage to cancer care. In addition, insurance should cover a comprehensive package of cancer-related services to enable holistic cancer prevention and care, including preventative services such as tobacco cessation and nutrition counseling, palliative care, ancillary services, and care delivery methods that facilitate access to care for individuals who face barriers to care (e.g., transportation and telehealth services), and survivorship services.

#### Recommendations for NCI

1. Create additional funding opportunities in the Division of Cancer Control and Population Sciences and the Center to Reduce Cancer Health Disparities to demonstrate the value of services that are critical to patient engagement in care, but rarely covered by traditional health insurance, especially among populations experiencing disparities, including transportation, nutrition support, navigation, and service delivery through telehealth.
2. Encourage cancer centers to establish partnerships with community organizations focused on policy advocacy and systems change that promotes cancer prevention.

#### Recommendations for cancer centers

1. Record and amplify patient and staff stories that illustrate areas where policy change is needed and partner with community-based organizations to leverage these stories for policy advocacy.
2. Educate policy-makers on gaps in care that result from lack of coverage or enrollment in health plans that do not comply with the Affordable Care Act Essential Health Benefit requirements, leaving patients underinsured.
3. Advocate for robust federal requirements for health insurance.

### Effectively evaluate COE

Currently in the COE mandate, cancer centers are required to demonstrate how they have addressed cancer health disparities and implemented health policy recommendations to decrease cancer incidence and mortality rates in their local communities. The full impact of COE efforts on cancer incidence and mortality will only be realized in the long term, yet COE is evaluated on a shorter time horizon.

Interim outcomes to measure progress on COE in the short and medium term are necessary to complement long-term impact outcomes. Cancer centers identified possible interim clinical and equitable care outcomes, such as change in stage of cancer at diagnosis, time between diagnosis and treatment, completing care, or change in racial/ethnic differences in cancer stage distribution. Participants also suggested possible complementary metrics that could evaluate the process and quality of COE, such as the number of individuals and community organizations reached through COE, the extent to which the community is involved in planning programs and designing studies, and changes in the capacity of community partner organizations to conduct research. Across these indicators, participants noted the importance of collecting both quantitative and qualitative inputs to inform assessments.

#### Specific recommendations for NCI

1. Update evaluation criteria to include interim, process-oriented metrics that can serve as signals for progress toward longer-term impact goals.
2. Engage experts with deep experience in the COE requirement areas to design relevant and realistic COE metrics. Engage reviewers with expertise and experience in the COE requirement areas to evaluate cancer center COE programs.
3. Create a process and structure for sharing research and COE results with the community.

#### Specific recommendations for cancer centers

1. Track short- and mid-term COE outcomes, disaggregated by subpopulation, to inform research and programming priorities.
2. Include community members in the development and interpretation of COE evaluation findings to ensure accurate representation and analysis, grounded in community impact.

## Conclusion

Cancer centers are increasingly seeing equity as a core aim of their work, and the NCI COE mandate has catalyzed additional investment. Challenges remain, however, to ensure COE activities result in sustained impact on population health. To improve equity in the system of cancer care, there is a need for improved understanding of health equity, local communities, and the factors that drive disparities. In addition, adequate resourcing and leadership commitment from the NCI and cancer centers, an ability to engage with partners within and outside of the heath sector to address structural barriers to care, including policy, and effective methods to track and evaluate progress toward health equity will be critical.

Recent experience with COE implementation has yielded lessons for how the NCI can be an effective partner for cancer centers working toward health equity. Continuing the dialog between the NCI, CCCs, professional and patient advocacy organizations, and communities can help shape NCI's COE priorities, mechanisms, and metrics to effectively achieve their intended impact of improved equity in cancer outcomes for communities and reduction of cancer burden.

## Abbreviations Used

AACR: American Association for Cancer Research
ACS: American Cancer Society
ASCO: American Society of Clinical Oncology
BMSF: Bristol Myers Squibb Foundation
CA: “catchment area”
CCC: Comprehensive Cancer Center
CCSG: Cancer Center Support Grants
CDC: Centers for Disease Control
COE: Community Outreach and Engagement
NCI: National Cancer Institute
SDOH: social determinants of health

## APPENDIX

### NCI CCCs, Affiliated Programs and Cancer Centers

- Carmen Guerra, *Abramson Cancer Center/University of Pennsylvania*
- Jenna Schiffelbein, *Dartmouth/Hitchock Cancer Center*
- Evelyn González, *Fox Chase Cancer Center*
- Martha Tingen, *Georgia Cancer Center, Augusta University*
- Mellisa Wheeler, *Levine Cancer Institute –Atrium Health*
- Neil Korsen*, Maine Medical Center*
- Ellen Baker, *MD Anderson Cancer Center—Project ECHO Cancer Super Hub*
- Francesca Gany, *Memorial Sloan Kettering Cancer Center*
- Lewis Kampel, *Ralph Lauren Center for Cancer Care*
- Anita Kinney, *Rutgers Cancer Institute of New Jersey*
- Ric Marlink*, Rutgers Global Health Institute*
- Grace Lu-Yao, *Sidney Kimmel Cancer Center/Jefferson Health*
- Monica Baskin, *University of Alabama at Birmingham O'Neal Cancer Center*
- Claudia Hardy, *University of Alabama at Birmingham O'Neal Cancer Center*
- Moon Chen, *University of California Davis Cancer Center*
- Sora Park Tanjasiri, *University of California Irvine Chao Family Comprehensive Cancer Center*
- Tung Nguyen, *UCSF Helen Diller Cancer Center*
- Neal Palafox, *University of Hawai'i Cancer Center*
- Karriem Watson, *University of Illinois Cancer Center*
- Vida Henderson, *University of Illinois Cancer Center*
- Jamie Studts, *University of Kentucky/Markey Cancer Center*
- Ursa Brown-Glaberman, *University of New Mexico Comprehensive Cancer Center*
- Andrew Sussman, *University of New Mexico Comprehensive Cancer Center*
- Oliver Bogler, *ECHO Institute/Project ECHO*
- Lucca Cirolia, *ECHO Institute/Project ECHO*
- Stephenie Kennedy-Rea, *West Virginia University Cancer Institute*
- Roy Herbst, *Yale Cancer Center*
- Beth Jones, *Yale Cancer Center*

### Cancer Professional Societies and Advocacy Groups

- Shakira Nelson, *American Association of Cancer Research*
- Christian Downs, *Association of Community Cancer Centers*
- Amanda Kramer, *Association of Community Cancer Centers*
- Ana Maria Lopez, *ASCO Health Equity Committee*
- Sarah Shafir, *American Cancer Society*
- Catharine Young, *Biden Cancer Initiative*
- Hildy Dillon, *Cancer Support Community*
- Jeanne Regnante, *National Minority Quality Forum*

### The National Cancer Institute

- Robert Croyle, *Division of Cancer Control and Population Sciences*
- Paul Jacobsen, *Division of Cancer Control and Population Sciences*
- LeeAnn Bailey, *Center to Reduce Cancer Health Disparities*
- Mary Ann Van Duyn, *Center to Reduce Cancer Health Disparities*
